# Bottom-up growth of n-type monolayer molecular crystals on polymeric substrate for optoelectronic device applications

**DOI:** 10.1038/s41467-018-05390-3

**Published:** 2018-07-26

**Authors:** Yanjun Shi, Lang Jiang, Jie Liu, Zeyi Tu, Yuanyuan Hu, Qinghe Wu, Yuanping Yi, Eliot Gann, Christopher R. McNeill, Hongxiang Li, Wenping Hu, Daoben Zhu, Henning Sirringhaus

**Affiliations:** 1 Beijing National Laboratory for Molecular Sciences, Key Laboratory of Organic Solids, Institute of Chemistry, Chinese Academy of Sciences, Zhongguancun North First Street 2, Beijing, 100190 China; 2 University of the Chinese Academy of Sciences, Beijing, 100049 China; 3 Cavendish Laboratory, Cambridge University, JJ Thomson Avenue, Cambridge, CB3 0HE UK; 4 Shanghai Institute of Organic Chemistry, Chinese Academy of Sciences, Shanghai, 200032 China; 5 Australian Synchrotron, 800 Blackburn Road, Clayton, VIC 3168 Australia; 6 Department of Materials Science and Engineering, Monash University, Wellington Road, Clayton, VIC 3800 Australia; 7 College of Science, Tianjin University, Tianjin, 300072 China

## Abstract

Self-assembly of monolayers of functional molecules on dielectric surfaces is a promising approach for the development of molecular devices proposed in the 1970s. Substrate chemically bonded self-assembled monolayers of semiconducting conjugated molecules exhibit low mobility. And self-assembled monolayer molecular crystals are difficult to scale up and limited to growth on substrates terminated by hydroxyl groups, which makes it difficult to realize sophisticated device functions, particularly for those relying on n-type electron transport, as electrons suffer severe charge trapping on hydroxyl terminated surfaces. Here we report a gravity-assisted, two-dimensional spatial confinement method for bottom-up growth of high-quality n-type single-crystalline monolayers over large, centimeter-sized areas. We demonstrate that by this method, n-type monolayer molecular crystals with high field-effect mobility of 1.24 cm^2^ V^−1^ s^−1^ and band-like transport characteristics can be grown on hydroxyl-free polymer surface. Furthermore, we used these monolayer molecular crystals to realize high-performance crystalline, gate-/light-tunable lateral organic p–n diodes.

## Abstract (web summary)

New methods for obtaining large-area monolayer molecular crystals (MMCs) on hydrophobic surfaces are needed to realize the full potential of MMCs for organic electronics. Here, the authors demonstrate bottom-up growth of high-grade n-type MMCs, which show superior performance in device applications.

## Introduction

Two-dimensional (2D) atomic materials have been intensely investigated since the emergence of graphene^1, 2^. Nowadays most of the research is focused on inorganic materials such as transition-metal dichalcogenides^3–5^, while only few studies of 2D organic semiconductors were reported because the growth of them is still challenging^6–9^. Scaling the long-range ordered 2D molecular crystals down to a thickness of one molecular layer provides great opportunities for fabricating high-performance monolayer transistors, which are expected to overcome the present mobility limitation of self-assembled monolayers (SAMs) below 0.05 cm^2^ V^−1^ s^−1^ ^10–13^ where the SAMs can be achieved by a substrate chemical bonding method^10–15^ or Langmuir–Blodgett method^16–19^. Monolayer transistors should enable very low contact resistance as no transport through the bulk is needed to access the charge accumulation layer at the interface between the SAM molecules and the dielectric. They also enable fundamental studies of the charge transport properties of organic molecules by providing direct access to probes, such as scanning Kelvin probe microscopy, to the accumulation layer at the interface^20^. However, one of the main challenges with building SAM transistors is that because the charge carriers are confined in the monolayer at the semiconductor-gate dielectric interface the charge transport processes are very sensitive to the surface of the dielectric^21^. For example, it was demonstrated that hydroxyl groups existing on the dielectric strongly inhibit n-channel filed-effect transistor (FET) conduction due to trapping of electrons at the semiconductor–dielectric interface^22^. To overcome this problem requires the ability to build SAM devices on a dielectric with low trap density. Quasi-free-standing monolayer molecular crystals (MMCs) open a way to construct monolayer semiconductors on hydroxyl-free surface, such as the divinyltetramethylsiloxane-*bis*(benzocyclobutene) derivative (BCB). However, among the limited 2D molecular crystals, MMCs that exhibit high carrier mobilities and intrinsic band-like temperature dependence of mobility are still scarce^6–8^.

Compared to thick crystals, MMCs also offer opportunities in bipolar devices such as gate-tunable p–n diodes as they allow confining the recombination regions to a quasi one-dimensional recombination zone between p-type and n-type molecular monolayers with potentially low losses. p–n junction diodes are essential building blocks of today’s optoelectronic device technology^23^. To the best of our knowledge, gate-tunable lateral p–n diodes based on organic crystals have not yet been demonstrated^5^. One of the main challenges for realizing lateral organic crystalline p–n junctions, compared to their inorganic counterparts, is the lack of techniques for lateral epitaxial growth or controlled in situ doping process of organic crystals. The availability of air-stable n-type MMCs and the ability to realize high-quality p–n contacts are the prerequisites for realizing such structures.

Here, we report an effective method for bottom-up growth of large area, air-stable n-type MMCs of conjugated molecule of dicyanomethylene-substituted fused tetrathienoquinoid (CMUT);^24^ and their use in high-performance n-type SAM transistors and lateral p–n diodes.

## Results

### Growth and characterization of MMCs of CMUT

**Fig. 1:**
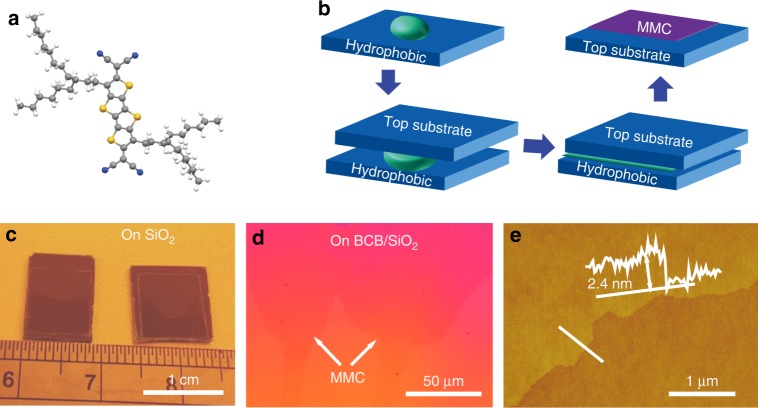
Schematic of CMUT molecule structure and preparation process of MMCs. **a** Molecular structure of CMUT. Gray represents carbon atom, yellow represents sulfur atom, blue represents nitrogen atom, and white represents hydrogen atom. **b** Schematic illustration of preparation of the MMCs. The semiconductor solution was dropped onto an OTS-treated SiO_2_/Si substrate, and then a top substrate was placed atop. Finally we obtained crystalline monolayer films on the top substrate after 24 h. **c** Large-area CMUT MMC up to centimeter size on SiO_2_/Si substrate. **d** Optical microscopy image and **e** corresponding AFM image of the MMC on BCB-treated SiO_2_/Si substrate

It is well known that organic semiconductors usually grow on SiO_2_-based substrates in a step-like, layer-by-layer growth mode^25–27^. In order to construct MMCs, the candidate molecules should have strong molecular interactions and orientation in 2D (not just 1D) within the intralayer, while a weak force along the interlayer direction is required to suppress the nucleation and growth of a second layer (Supplementary Fig. 1). According to this strategy, we use CMUT as a candidate semiconductor (Fig. 1a). Within the intralayer we expect 2D S···N non-bonded contacts and short *π*–*π* stacking, as deduced from the molecular structure of a related compound with the same core, dicyanomethylene-substituted tetrathienoquinoid with hexyl substituents (CMHT)^28^. Despite focused attempts, we were unable to grow CMUT single crystals with sufficient size for crystal structure determination, while CMHT readily formed large single crystals with submillimeter dimensions. The introduction of the branched alkyl chain in CMUT compared to CMHT is expected to weaken interlayer forces by suppressing attractive N···H interactions along the interlayer direction and also improve the solubility of the compound in common organic solvents for solution-processing. In this way, an efficient 2D intralayer preferential molecular stacking could be realized for the growth of large MMCs (Supplementary Fig. 2).

To prepare the MMCs a gravity-assisted 2D spatial confinement method was developed as shown in Fig. 1b. Overall, 5–30 μL of chlorobenzene solution of CMUT (2 mg/mL) was dropped onto a hydrophobic substrate (octadecyltrichlorosilane (OTS)-treated SiO_2_/Si wafers), which then was covered by a hydrophilic SiO_2_/Si substrate. As the solvent evaporates, a very thin layer of solution is formed in the 2D space between the two substrates assisted by the gravity of the top substrate, thus leading to the growth of MMCs on the top hydrophilic substrate after 24 h. The better wetting properties of chlorobenzene on the top substrate than that on the bottom substrate probably induce the selected growth of the MMCs on the top substrate (Supplementary Fig. 3). Typical optical images of the MMCs are presented in Fig. 1c (centimeter size). It is clear that a uniform monolayer was obtained (Supplementary Fig. 4). To our surprise, when a BCB-treated SiO_2_/Si wafer was used as the top substrate instead of the bare SiO_2_/Si substrate, MMCs were grown on the top substrate as well (Fig. 1d). The thickness of the CMUT film is about 2.4 nm (Fig. 1e), which would be one molecular layer corresponding to calculated single molecular length. To our knowledge, it is for the first time ever that MMCs are successfully prepared on polymer substrates.

**Fig. 2:**
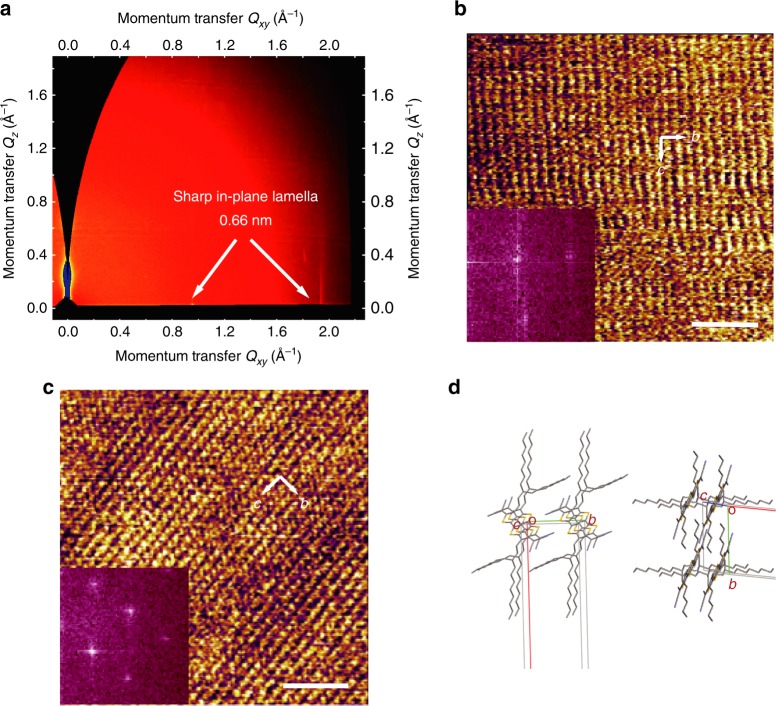
Structural characterizations of the monolayer of CMUT on SiO_2_/Si^++^. **a** The GIWAXS pattern of the monolayer. **b**, **c** HR-AFM image of the monolayer, here the scale bar is 5 nm, **b** top side (*b* = 8.3 Å, *c* = 6.5 Å, *θ* = 90°) and **c** bottom side (*b* = 8.4 Å, *c* = 6.5 Å, *θ* = 91°). **d** Monolayer crystal structure obtained by full optimization at the DFT-D3 level (*b* = 9.9 Å, *c* = 5 Å, *θ* = 101.6°)

To gain insights into the molecular arrangement of the crystalline film, structure characterization by Grazing-Incidence Wide-Angle X-ray Scattering (GIWAXS) was performed on monolayer films and on thicker crystalline reference films. For the monolayer, we detected a faint out-of-plane diffraction peak corresponding to a lamella thickness of about 1 nm and a sharp in-plane diffraction peak corresponding to a molecular distance of 0.66 nm (Fig. 2a). The scattering pattern is typical of high-quality self-assembled monolayers. The corresponding 1D plots shown in Supplementary Fig. 5 provide evidences for a high degree of structural order within the molecular layer with long coherence length of 30 nm that can be observed despite the overall weak signal due to the ultra-thin film thickness. Interestingly, the GIWAXS pattern of the thick film (Supplementary Fig. 6) is different from that of the monolayer. The optimized monoclinic lattice parameters extracted from such patterns are *a* = 2.54 nm, *b* = 1.12 nm, *c* = 0.51 nm, *α* = 75.6°, *β* = 85.3°, *γ* = 90.7°. In *θ*-2*θ* scans many areas of the sample show only the *h*00 peaks (Supplementary Fig. 7), indicating a layer-by-layer growth with a different layer structure to that found in the monolayer film. From this, we would expect to observe diffractions corresponding to the *b*–*c* crystalline plane for in-plane scattering geometry. These do not match the 0.66 nm periodicity that is observed in the monolayers. Further structure characterization was performed by high-resolution atomic force microscope (HR-AFM) on monolayer and thick films. The lattice constants along the *b* and *c* axes are ~0.83 ± 0.01 and ~0.65 ± 0.02 nm, respectively, for the monolayer, while ~1.04 ± 0.01 and ~0.52 ± 0.01 nm for the thick films (Fig. 2b, Supplementary Fig. 8). This is fully consistent with the GIWAXS results. In order to verify that only a quasi-free-standing MMCs without embedded interfacial layer was grown on the substrates, HR-AFM was also carried out on the bottom side of the MMCs by utilizing a peeling off processes (Supplementary Figs. 9, 10). Both top side and bottom side of the MMCs exhibit indeed a very similar packing mode (Fig. 2b, c). HR-AFM measurements were also performed across a large area of the MMC. The same lattice structures (without rotation) were obtained when the AFM scanned across length scales of several hundred micrometers, which demonstrates that over this length scale the large-area MMC is likely to be a single crystal (Supplementary Fig. 11). Note that a very similar molecular packing mode of the MMCs is observed on both SiO_2_ and BCB/SiO_2_ surface (Supplementary Fig. 12). We only observed a slightly larger thickness of the MMCs on BCB-treated substrate, the thickness is about 2 nm on SiO_2_ (Supplementary Fig. 4b) and 2.4 nm on BCB/SiO_2_ surface (Fig. 1e). It suggests that the CMUT molecules stand on the BCB/SiO_2_ surface with a slightly higher tilt angle than those on SiO_2_. We find that both the monolayer and thick films are highly crystalline with typical brick-wall-like packing in the (100) plane through fast Fourier transformation (FFT) of HR-AFM images. Raman spectrum measurement was also performed to crosscheck the difference of the monolayer and thick film (Supplementary Fig. 13). We can see some obviously different peaks between the monolayer and thick film phases, which further confirms the different phases between the monolayer and thick films. Moreover, the bulk and monolayer crystal structures were simulated by combining molecular mechanics (MM) and density functional theory including D3 dispersion correction (DFT-D3). Firstly, the atomic positions in the unit cell were determined by MM based on the experimentally measured cell parameters. Then the crystal structures were optimized by DFT-D3 with the cell parameters relaxed or not (see Supplementary Fig. 25). We note that for the bulk crystal of CMUT, the cell parameters of the fully optimized crystal (*a* = 24.75 Å, *b* = 10.56 Å, *c* = 5.05 Å, *α* = 73.5°, *β* = 98.7°, *γ* = 97.2°) are very similar to the experimental data, especially for the cell parameters relevant to the 2D molecular layer (*b*, *c*, and *α*). This means it is feasible to theoretically determine the crystal molecular packing according to the experimentally measured cell parameters. Due to the lack of substrates in our simulations, the fully optimized cell parameters for the MMC (*b* = 9.92 Å, *c* = 5.04 Å, *θ* = 101.6°) vary much from the experimentally measurements, indicating an important role the substrates play in preparation of the monolayer crystal.

### MMC FETs of CMUT

**Fig. 3:**
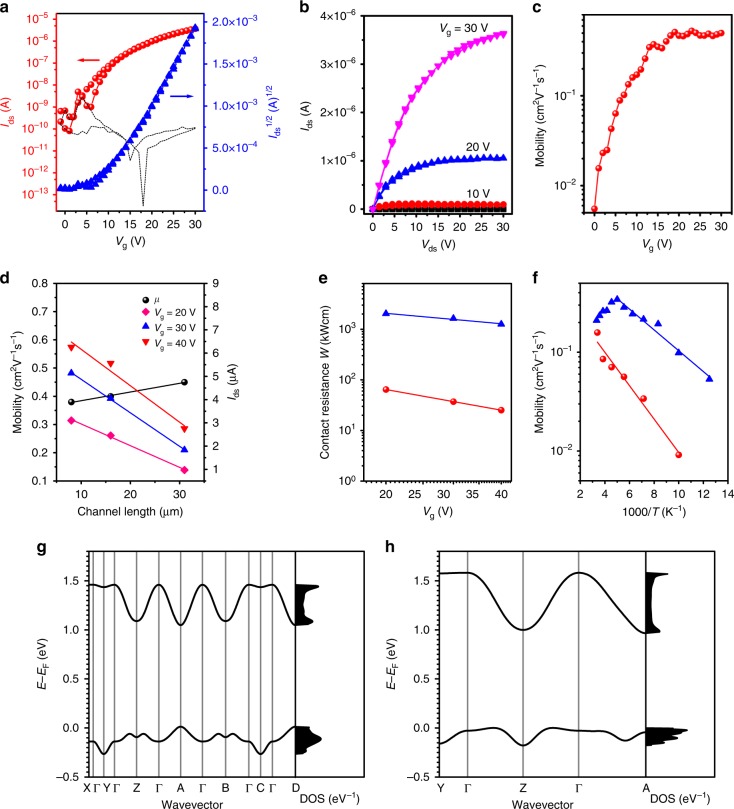
Electrical properties of MMC FETs. **a**, **b** Transfer and output curves of MMC devices on BCB/SiO_2_ substrates (in **a**, the dashed black line is the leakage current). Here the channel width and length are 131 and 19 μm, respectively, and the saturation mobility is 0.51 cm^2^ V^−1^ s^−1^. **c** The extracted field-effect mobility as a function of *V*_g_ at *V*_ds_ = 30 V for the same device. **d** Electron mobility and drain current of the MMC FETs at different channel lengths on BCB/SiO_2_. **e** Contact resistance of MMC FETs (red ball) and FETs based on the thick crystal with thickness of 40 nm (blue triangle) at different gate voltages on BCB/SiO_2_. **f** Temperature-dependent mobility of the MMC on SiO_2_ (red ball) and BCB/SiO_2_ (blue triangle). **g**, **h** Band structures and density of states (DOS) of the fully optimized bulk (**g**) and monolayer crystals (**h**). The high-symmetry points in the first Brillouin zone are labeled as follows: *Г* = (0,0,0), *X* = (0.5,0,0), *Y* = (0,0.5,0), *Z* = (0,0,0.5), *A* = (0,0.5,0.5), *B* = (0.5,0,0.5), *C* = (0.5,0.5,0), and *D* = (0.5,0.5,0.5), all in crystallographic coordinates. The Fermi energy is taken as the origin of the energy axis

The electron mobility was measured in air employing a bottom-gate, top-contact FET configuration with Au stripes acting as source and drain electrodes^29^. The average saturation mobility of the MMC is 0.12 cm^2^ V^−1^ s^−1^ on bare SiO_2_ substrates (Supplementary Fig. 14). In comparison, the average saturation mobility value of the MMC FETs on BCB/SiO_2_ substrate is ~0.5 cm^2^ V^−1^ s^−1^ with maximum value up to 1.24 cm^2^ V^−1^ s^−1^ (Supplementary Fig. 15) which are comparable to or even higher than those of thick films (the average saturation mobility is ~0.39 cm^2^ V^−1^ s^−1^ when the thickness is about 40 nm, as shown in supplementary Fig. 16). Typical electrical characteristic curves of the MMC FETs on BCB/SiO_2_ are shown in Fig. 3a, b, the saturation mobility is 0.51 cm^2^ V^−1^ s^−1^ (the linear mobility is about 0.52 cm^2^ V^−1^ s^−1^, which is close to the saturation mobility providing evidence for a low contact resistance. See Supplementary Figs. 17, 18). It is also observed that the devices on BCB/SiO_2_ substrates exhibit no obvious hysteresis and are more stable than those on bare SiO_2_ substrates in air. The mobility is seen to be almost gate-independent when *V*_g_ is above the threshold voltage *V*_th_ (about 10 V), as shown in Fig. 3c, indicating that the effects of contact resistance and interface traps on the device performance are negligible^30^. We also measured MMC FETs with different channel lengths realized by immobilizing source Au electrode and moving the drain Au electrodes (Supplementary Figs. 19, 20). The mobility as a function of channel length is shown in Fig. 3d. There is no strong degradation of electron mobility with the decrease of channel length, which verifies the high quality of the MMCs with long-range order molecular packing and low access/contact resistance in the MMC-based devices. Meanwhile, the current is linearly increased with the decrease of channel length (Fig. 3d), which is caused by the uniformly decrease of channel resistance in the MMC device. As shown in output curve, the MMC FETs exhibit more linear characteristics at low *V*_ds_ voltages compared to the thick crystal FETs, which demonstrates that MMC FETs show lower contact resistance than the FETs based on thick films (Fig. 3b and Supplementary Fig. 16b). By employing a conventional transfer line method (TLM), we extracted the value of the contact resistance (*R*_C_) in FET devices. We find that values of *R*_C_ in monolayer FETs are over 30 times smaller than those of FETs based on the thick crystals (Fig. 3e, Supplementary Fig. 21). This clearly demonstrates the low access/contact resistance advantage that MMC-based devices have over thicker film-based devices.

The current–voltage curves of devices on both SiO_2_/Si and BCB/SiO_2_ substrates were then measured at different temperatures, and the mobility as a function of temperature was extracted, as shown in Fig. 3f. Activation energy *E*_a_ = 33.6 meV and 20.7 meV were extracted for SiO_2_/Si and BCB/SiO_2_-based devices, respectively, calculated by fitting the low temperature behavior below 200 K to *μ* ∝ exp (−*E*_a_/*k*_B_*T*) with the Boltzmann constant *k*_B_ and temperature *T*. The value of *E*_a_ on BCB/SiO_2_ is much lower than the values reported previously in high-performance organic semiconductors^31, 32^. Moreover, the mobility of the MMC devices based on the BCB/SiO_2_ substrate decreased with increasing temperature between 200 and 300 K, which indicates that band-like transport characteristics can be observed in this temperature range. In comparison, such band-like behavior was not seen in the devices prepared on SiO_2_ substrates. To the best of our knowledge, a band-like temperature dependence of the mobility has not yet been demonstrated before in organic monolayer transistors. This difference in the two types of devices can possibly be accounted for by two reasons. First, the density of deep electron traps at the BCB/MMC interface is lower than that at the SiO_2_/MMC interface, which is favorable for the appearance of band-like transport. Second, it is known that the dipoles in the dielectrics can introduce disorder in the transport layers. The dipole-induced disorder plays an important role in determining the temperature-dependent properties^33, 34^. Considering that BCB has a much lower dielectric constant (2.6) than SiO_2_ (3.9), a lower dipole-induced disorder is thus expected in the devices on BCB/SiO_2_ substrates, making it more likely to observe more intrinsic band-like transport characteristics. Furthermore, the conduction band along the stacking direction of the MMC was calculated to be very dispersive (the band width is at least 600 meV and even larger than that of the bulk crystal), leading to a small effective mass for electron transport (0.70 *m*_0_ or smaller) (see Fig. 3g, h and Supplementary Fig. 27 and Supplementary Table 3). These calculated results are consistent with the observation of band-like transport in the MMCs.

### Applications on p–n diode of CMUT MMCs

**Fig. 4:**
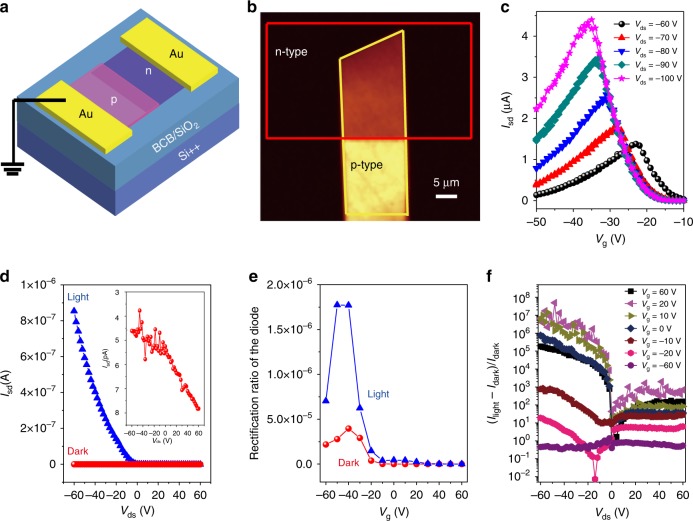
Optoelectronic characteristics of lateral organic p–n diode. **a** Schematic diagram of lateral organic p–n diode. The electrode at the p-type side was set grounded. **b** Fluorescent mapping of p–n diode. **c** Transfer curves of the p–n diode at *V*_ds_ = −60 to −100 V measured in the dark. **d** Output characteristics of the p–n diode as gate bias voltage at *V*_g_ = 60 V under light illumination (blue triangle) and in the dark (red ball) condition. The inset shows the zoom in curves in the dark condition. **e** Rectification curve of the p–n diode at *V*_ds_ = ±60 V. Blue triangle is under light illumination and red ball is in the dark condition. **f** Photosensitivity of the p–n diode at different gate voltages

We then fabricated lateral p–n junctions by mechanically transferring a p-type 2,6-diphenylanthracene (DPA) crystal (the thickness is about 20 nm) onto the n-type MMC (Supplementary Fig. 22). A schematic diagram of the p–n junction is shown in Fig. 4a. CMUT is a non-fluorescent molecule, but DPA is highly fluorescent^35^, this enabled fluorescence mapping across the p–n junction. As shown in Fig. 4b, the dim region of the junction reflects the area of the junction in which the n-type and p-type material overlap and in which the luminescence from the DPA is efficiently quenched by charge transfer to the CMUT. The bright region is the one in which only the p-type material is present. The fluorescence map demonstrates an abrupt p–n junction. The homogeneous brightness of the overlap region indicates the excellent uniformity of the MMC. However, thick films failed in constructing lateral p–n junctions. When thick CMUT films with thickness larger than 30 nm were used, the p-type semiconductor DPA crystals were found to crack at the edge of the junction (Supplementary Fig. 23). When measured under forward bias (negative *V*_d_) in the dark the p–n junctions based on n-type MMCs show a clear gate-tunable drain current characteristics (Fig. 4c), which is expected for such p–n junctions between two unipolar materials. Taking the curve at the drain voltage of −60 V, for example, the current is low at negative gate voltages (*V*_g_ near −10 V). In this region, electrons are accumulated in CMUT, but a hole accumulation layer is not yet formed in the DPA, i.e., the current flow is limited by the high resistance of the DPA crystal. Likewise, at *V*_g_ near −60 V the hole accumulation layer in the DPA crystal is fully formed, but there is now no electron accumulation in the CMUT and as a result the current is low again. In the intermediate voltage regime, the current reaches a maximum near |*V*_g_| ≈ |*V*_d_|/2; in this regime electrons are injected from the drain contact into the electron accumulation layer in the CMUT, while holes are injected from the source contact into the hole accumulation layer in DPA. Under these biasing conditions, the peak in the current reflects the recombination current at the heterointerface. However, we were not able to observe any electroluminescence from the junction, suggesting that the recombination is non-radiative. This is consistent with the energy levels of the two materials: the LUMO and HOMO levels of DPA are 2.6 and 5.6 eV, respectively, while the corresponding levels for CMUT are 4.3 and 6.1 eV. This suggests that recombination is more likely to occur in the (non-luminescent) CMUT as the hole injection barrier from DPA into CMUT is expected to be significantly smaller than the electron injection barrier from CMUT into DPA.

When measuring the current as a function of *V*_ds_ for a given *V*_g_, we observe strongly rectifying characteristics with the current being significantly higher in forward bias (negative *V*_ds_) than in reverse bias. We also observe the current depends strongly on light illumination (Supplementary Fig. 22). In the dark, the forward current depends strongly on gate voltage; the current is very small for large, positive *V*_g_ and much larger for negative *V*_g_, suggesting that in the dark the forward current might be limited by the current transport through the p-type DPA crystal. Under light, the current depends much less strongly on gate voltage, but is increased at larger positive *V*_g_. This suggests that under light the current is limited by transport through the CMUT crystal. Figure 4d shows the typical output characteristic curves of the p–n junctions at *V*_g_ = 60 V. The red ball curve is in the dark condition and the blue triangle is under light illumination. The inset in Fig. 4d shows a zoomed-in graph of the dark current at *V*_g_ = 60 V. Figure 4e shows the rectification curve of the p–n diode, here the rectification ratio is defined as the ratio of the current at the maximum forward bias to the current at the same magnitude reverse bias. The maximum rectification ratios are 4 × 10^5^ and 1.8 × 10^6^ in the dark condition and under light illumination, respectively, which are significantly higher than the previously reported values to date^36^. In the dark state, the diodes exhibit a wide tunable range of rectification ratios from 0.6 to 4 × 10^5^. The photosensitivity is defined as *P* = (*I*_light_ − *I*_dark_)/*I*_dark_, here *I*_dark_ and *I*_light_ are the current in the dark condition and under light illumination, respectively. The photosensitivity of the p–n diode at different gate voltages is shown in Fig. 4f, which exhibits high photosensitivity up to 10^7^. The photosensitivity is highest in forward bias in a gate voltage regime (*V*_g_ = 10–20 V), where the CMUT is expected to be electron accumulated but the DPA crystals are not yet expected to be hole accumulated. However, even in reverse bias where the CMUT tends to be depleted a photosensitivity up to 10^3^ is achievable. The optoelectronic measurements conclusively demonstrate that our MMC p–n diodes exhibit high-performance gate-tunable and light-tunable electrical characteristics, which is comparable to their counterpart based on the atomically thin inorganic 2D materials^5, 37^.

## Discussion

In conclusion, large-area n-type MMCs were successfully prepared by a gravity-assisted 2D spatial confinement method on SiO_2_/Si and polymer substrate. MMC FETs show high mobility up to 1.24 cm^2^ V^−^^1^ s^−1^ with negligible channel length dependence. The high-performance MMC FETs provide a great platform for performing charge transport studies. The MMC transistors based on BCB/SiO_2_ substrates exhibit band-like transport (d*µ*/d*T* < 0) at temperatures above 200 K, and thermally activated transport with low thermal activation energy of about 20.7 meV from 80 to 200 K, demonstrating the high quality of the MMCs. We then realized a high-performance gate-/light-tunable lateral organic crystalline p–n junction based on the MMCs. The rectification ratios are 4 × 10^5^ and 1.8 × 10^6^ in the dark condition and under light illumination, respectively, and the photosensitivity is up to 10^7^, which are significantly higher than the previously reported values to date. Our facile method for growing MMCs on trap-free, polymeric surface is promising to the preparation of a wide range of materials that meet the molecular design requirements for MMCs outlined above (Supplementary Fig. 24). The high performance of our simple MMC-based p–n junction suggests that our method should allow realization of a wide range of more complex device architectures for fundamental studies of device physics and for a range of optoelectronic applications as efficient rectifiers and photodetectors or low-dimensional light emitters or highly sensitive sensors.

## Methods

### Growth and characterization of CMUT MMCs

The MMC was prepared with a method that 5–30 µL chlorobenzene solution of 2 mg mL^−1^ CMUT molecule materials was dropped onto an OTS-treated SiO_2_/Si substrate (0.8 × 0.8 cm^2^), and then covered by a bare SiO_2_/Si substrate (1 × 1 cm^2^). We also obtained the MMC on BCB-treated SiO_2_/Si substrate. Contact angles were tested on a Contact Angle Meter (DSA 100, KrÜss Company, Germany) by a sessile droplet method. Saturation and linear mobility can be estimated using the equation: ${\mathrm{\mu }}\, = \,\frac{{2L}}{{WC_i}}(\frac{{\partial \sqrt {I_{{\mathrm{ds}}}} }}{{\partial V_{\mathrm{g}}}})^2$ and $\mu \, = \frac{L}{{WC_iV_{{\mathrm{ds}}}}}\frac{{\partial I_{{\mathrm{ds}}}}}{{\partial V_{\mathrm{g}}}}$, respectively. Here, *L* and *W* are the channel length and width of devices, respectively, $C_i$ is capacitance of the gate-dielectric capacitance per unit area. The capacitance characteristic was measured with Princeton Applied Research. The morphology was characterized by optical microscope. The thickness of the crystalline films was confirmed by atomic force microscope. The structures were characterized by GIWAXS, high-resolution AFM and Raman spectrum. DPA crystals were achieved by means of physical vapor transport (PVT) (165 °C, 5 h, 20 Pa, under argon atmosphere (0.04 L min^−1^)). The p–n junction was fabricated by using cantilever probes to mechanically transfer crystalline DPA onto the MMC. HR-AFM were performed by Cypheres. Optoelectronic characteristics were measured by using a Keithley 4200-SCS semiconductor parameter analyzer and a Micromanipulator 6150 probe station in a clean and shielded box in the ambient environment at room temperature in the dark condition and under white light illumination (0.4 mW cm^−2^).

### Details of DFT calculations

To simulate the bulk crystal structures, we assume that CMUT has similar packing mode as the CMHT crystal, which exhibits strong 2D packing (see Supplementary Fig. 2) and $P\bar 1$ space groups. According to the experimental unit cell of CMUT, we constructed initial geometries to form 2D packing without close contacts through carefully tuning the *π*–*π* slips of backbones and the orientations of alkyl chains, and optimized the atomic positions by using molecular mechanics with the compass force field. Subsequently, the crystal structures were optimized by DFT with the HSE06 functional including the Grimme D3 dispersion correction (DFT-D3) ^38, 39^. Finally, both the atomic positions and cell parameters were fully optimized at the DFT-D3 level (denoted as fullopt). The Monkhorst–Pack *k*-point meshes for Brillouin zone integration were set to 1 × 2 × 5 for geometry optimizations and 1 × 4 × 9 for the calculations of band structures and density of states (see Supplementary Figs. 26, 27 and Supplementary Tables 1, 2, 3 and Supplementary Note 1: Computational details for transfer integrals and reorganization energies).

Based on the (100) surface of the simulated bulk crystal structure and the experimental cell parameters of the MMC, we first created an enlarged monolayer molecular crystal (*b* = 20 Å, *c* = 20 Å, *θ* = 88°, and 60 Å for the non-periodic direction), and then repeatedly (20 times in total) reduced the lattice constants toward the experimental data; during this process, the atomic positions were optimized by using the compass force field. At last, the atomic positions of the MMC were further optimized at the DFT-D3 level with the cell parameters constrained or relaxed (fullopt). The Monkhorst–Pack *k*-point meshes were set to 1 × 3 × 4 for geometry optimizations and 1 × 5 × 8 for the calculations of band structures and density of states. The inverse effective mass tensor was calculated from the band structure by using Sperling’s centered difference method with *dk* = 0.01 Bohr^−1^. Subsequent diagonalization of $m_{ij}^{ - 1}$ provided the principal components and their orientations. All the above DFT calculations adopted the basis set of def2-SVP for S and 6–31G* for the other atoms and were carried out with the CRYSTAL17 package^40^.

### Data availability

The data that support the findings of this study are available from the corresponding author on reasonable request.

## Electronic supplementary material

Supplementary Information

## References

[1] Novoselov KS (2015). Two-dimensional atomic crystals. Proc. Natl Acad. Sci. USA.

[2] Coleman JN (2011). Two-dimensional nanosheets produced by liquid exfoliation of layered materials. Science.

[3] Duan XD, Wang C, Pan AL, Yu RQ, Duan XF (2015). Two-dimensional transition metal dichalcogenides as atomically thin semiconductors: opportunities and challenges. Chem. Soc. Rev..

[4] Chhowalla M (2013). The chemistry of two-dimensional layered transition metal dichalcogenide nanosheets. Nat. Chem..

[5] Lee CH (2014). Atomically thin p–n junctions with van der Waals heterointerfaces. Nat. Nanotechnol..

[6] Jiang L (2011). Millimeter-sized molecular monolayer two-dimensional crystals. Adv. Mater..

[7] He DW (2014). Two-dimensional quasi-freestanding molecular crystals for high-performance organic field-effect transistors. Nat. Commun..

[8] Zhang YH (2016). Probing carrier transport and structure-property relationship of highly ordered organic semiconductors at the two-dimensional limit. Phys. Rev. Lett..

[9] Xu CH (2016). A general method for growing two-dimensional crystals of organic semiconductors by “solution epitaxy”. Angew. Chem. Int. Ed..

[10] Smits ECP (2008). Bottom-up organic integrated circuits. Nature.

[11] Mathijssen SG (2009). Monolayer coverage and channel length set the mobility in self-assembled monolayer field-effect transistors. Nat. Nanotechnol..

[12] Novak M (2011). Low-voltage p- and n-type organic self-Assembled monolayer field effect transistors. Nano Lett..

[13] Ringk A (2013). n-Type self-assembled monolayer field-effect transistors for flexible organic electronics. Org. Electron..

[14] Guo XF (2006). Chemoresponsive monolayer transistors. Proc. Natl Acad. Sci. USA.

[15] Tulevski GS (2004). Attaching organic semiconductors to gate oxides: in situ assembly of monolayer field effect transistors. J. Am. Chem. Soc..

[16] Alexey SS (2013). Oligothiophene-based monolayer field-effect transistors prepared by Langmuir–Blodgett technique. Appl. Phys. Lett..

[17] Alexey SS (2014). Easily processable highly ordered Langmuir–Blodgett films of quaterthiophene disiloxane dimer for monolayer organic field-effect transistors. Langmuir.

[18] Borshchev OV (2017). Synthesis of organosilicon derivatives of [1]benzothieno[3,2-b][1]-benzothiophene for efficient monolayer Langmuir–Blodgett organic dield effect transistors. Chem. Comm..

[19] Elena, V. A. et al. Luminescent organic semiconducting Langmuir monolayers. *ACS Appl. Mater. Interfaces* **9**, 18078–18086 (2017). 10.1021/acsami.7b01919 28488872

[20] Hu YY (2016). Scanning Kelvin probe microscopy investigation of the role of minority carriers on the switching characteristics of organic field-effect transistors. Adv. Mater..

[21] Kobayashi S (2004). Control of carrier density by self-assembled monolayers in organic field-effect transistors. Nat. Mater..

[22] Chua LL (2005). General observation of n-type field-effect behaviour in organic semiconductors. Nature.

[23] Liu J, Engquist I, Crispin X, Berggren M (2012). Spatial control of p–n junction in an organic light-emitting electrochemical transistor. J. Am. Chem. Soc..

[24] Wu QH (2011). Dicyanomethylene-substituted fused tetrathienoquinoid for high-performance, ambient-stable, solution-processable n-channel organic thin-film transistors. Chem. Mater..

[25] Jiang L, Hu WP, Wei ZM, Xu W, Meng H (2009). High-performance organic single-crystal transistors and digital inverters of an anthracene derivative. Adv. Mater..

[26] Virkar AA, Mannsfeld S, Bao ZN, Stingelin N (2010). Organic semiconductor growth and morphology considerations for organic thin-film transistors. Adv. Mater..

[27] Liu SH, Wang WM, Briseno AL, Mannsfeld SCB, Bao ZN (2009). Controlled deposition of crystalline organic semiconductors for field-effect-transistor applications. Adv. Mater..

[28] Wu QH (2014). High-performance n-channel field effect transistors based on solution-processed dicyanomethylene-substituted tetrathienoquinoid. RSC Adv..

[29] Tang QX (2008). High-performance air-stable bipolar field-effect transistors of organic single-crystalline ribbons with an air-gap dielectric. Adv. Mater..

[30] Choi HH, Cho K, Frisbie CD, Sirringhaus H, Podzorov V (2018). Critical assessment of charge mobility extraction in FETs. Nat. Mater..

[31] Aleshin AN, Lee JY, Chu SW, Kim JS, Park YW (2004). Mobility studies of field-effect transistor structures basedon anthracene single crystals. Appl. Phys. Lett..

[32] Kim GT (2000). Field-effect transistor made of individual V_2_O_5_ nanofibers. Appl. Phys. Lett..

[33] Coropceanu V, Bredas JL (2006). Organic transistors: a polarized response. Nat. Mater..

[34] Minder NA, Ono S, Chen ZH, Facchetti A, Morpurgo AF (2012). Band-like electron transport in organic transistors and implication of the molecular structure for performance optimization. Adv. Mater..

[35] Liu J (2015). High mobility emissive organic semiconductor. Nat. Commun..

[36] Dhar BM (2010). Field-effect-tuned lateral organic diodes. Proc. Natl Acad. Sci. USA.

[37] Huo NJ (2015). Novel optical and electrical transport properties in atomically thin Wse_2_/MoS_2_ p–n heterostructures. Adv. Electron. Mater..

[38] Grimme S (2010). A consistent and accurate ab initio parametrization of density functional dispersion correction (DFT-D) for the 94 elements H-Pu. J. Chem. Phys..

[39] Moellmann J (2014). DFT-D3 study of some molecular crystals. J. Phys. Chem. C..

[40] Erba A (2017). Large-scale condensed matter DFT simulations: performance and capabilities of the CRYSTAL code. J. Chem. Theory Comput..

