# Supplementary material for: Bottom-up growth of n-type monolayer molecular crystals on polymeric substrate for optoelectronic device applications
Source: Nat Commun. 2018 Jul 26;9:2933. doi: 10.1038/s41467-018-05390-3 (PMC6062560; doi:10.1038/s41467-018-05390-3)
Supplement: Supplementary file 1 — Supplementary Information [file 41467_2018_5390_MOESM1_ESM.pdf]

Bottom-up growth of n-type monolayer molecular crystals on polymeric substrate for optoelectronic device applications

Shi *et al*.

Bottom-up growth of n-type monolayer molecular crystals on polymeric substrate for optoelectronic device applications

Yanjun Shi, Lang Jiang, Jie Liu, Zeyi Tu, Yuanyuan Hu, Qinghe Wu, Yuanping Yi, Eliot Gann, Christopher R. McNeill, Hongxiang Li, Wenping Hu, Daoben Zhu, Henning Sirringhaus

Supplementary Figures:

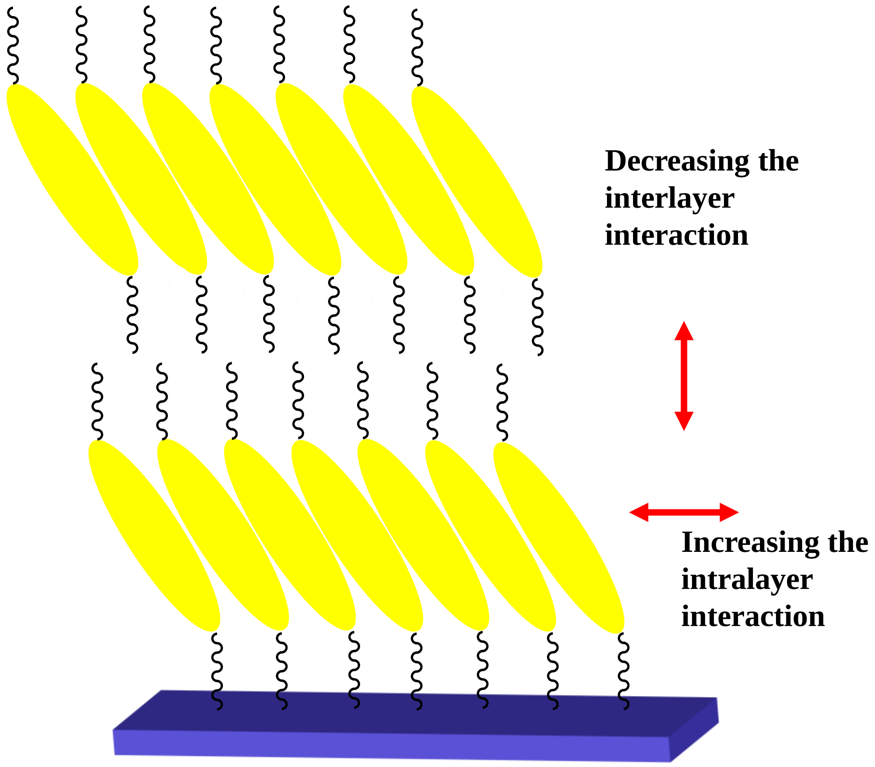

Supplementary figure 1 | The strategy of constructing MMCs (monolayer molecular crystals). As well known, organic semiconductors usually grow on SiO_2 based substrates in a step-like, layer-by-layer growth mode. In order to construct MMCs, the candidate molecules should have strong molecular interactions and orientation in 2D (not just 1D) within the intralayer, while a weak force along the interlayer direction is required to suppress the nucleation and growth of a second layer.

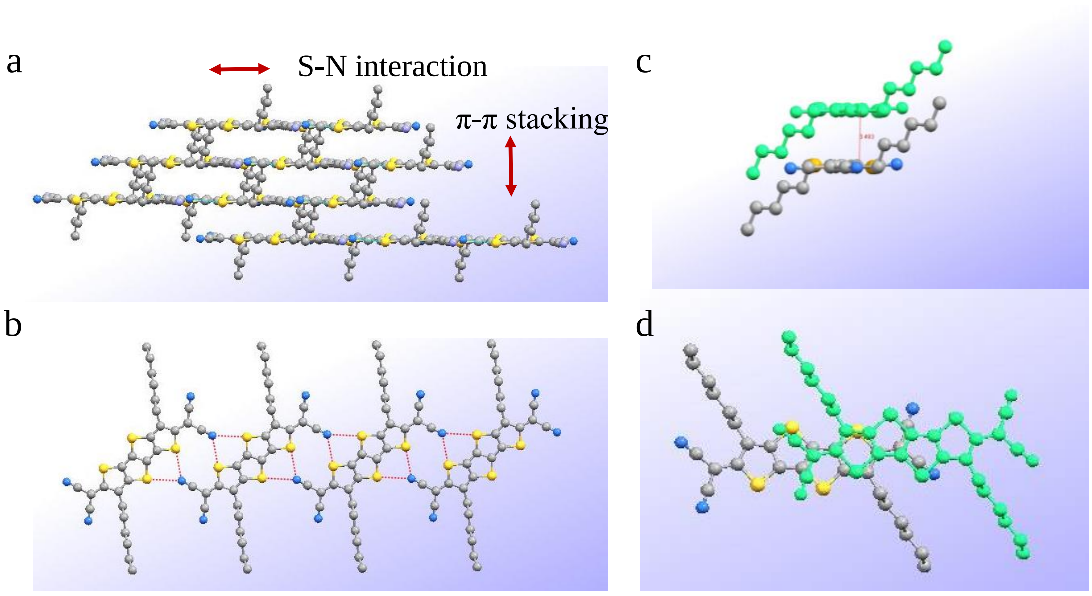

Supplementary Figure 2 | The molecular packing structures of CMHT (dicyanomethylene-substituted tetrathienoquinoid with hexyl substituents). a, Strong 2D intralayer interaction. In the crystal structure of a related compound with the same core, CMHT, the non-bonded contacts (S···N) and short π-π stacking distance induce strong 2D intralayer interaction. **b,** The S···N interactions within the intralayer. The introduction of the branched alkyl chain in CMUT (dicyanomethylene-substituted fused tetrathienoquinoid) is expected to prevent the force of interlayer interaction and also improve the solubility of the compound in common organic solvents for solution processing. **c,** Side view and d, top view of π-π stacking. The distance is about 0.35 nm.

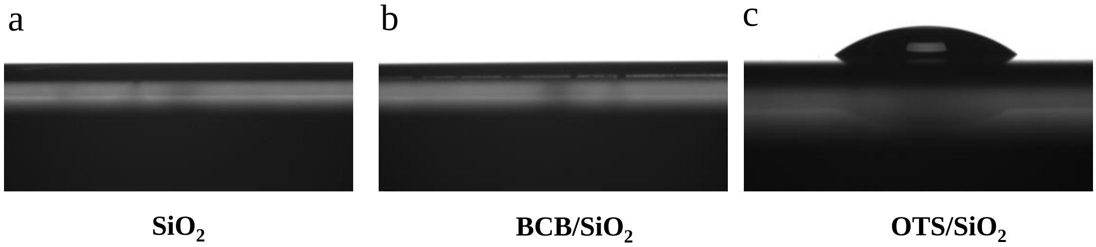

Supplementary Figure 3 | Contact angle tests of chlorobenzene on different substrates. a, SiO_2/Si **b,** BCB/SiO_2/Si, **c,** OTS-SiO_2/Si. In the gravity-assisted 2D spatial confinement method, chlorobenzene solution of CMUT was dropped onto a hydrophobic substrate (OTS-treated SiO_2/Si wafers), which then was covered by a hydrophilic SiO_2/Si substrate, thus leading to the growth of MMCs on the top hydrophilic substrate. If the top substrate is hydrophobic BCB-treated SiO_2/Si wafers, we also obtained the MMCs grown on top substrates. This should be attributed to the more excellent wetting properties of chlorobenzene on top substrates than that on bottom substrates. Here, the contact angle of chlorobenzene on OTS is about 37.1^{o}, which is much higher than that on SiO_2/Si and BCB/SiO_2/Si (about 0°).

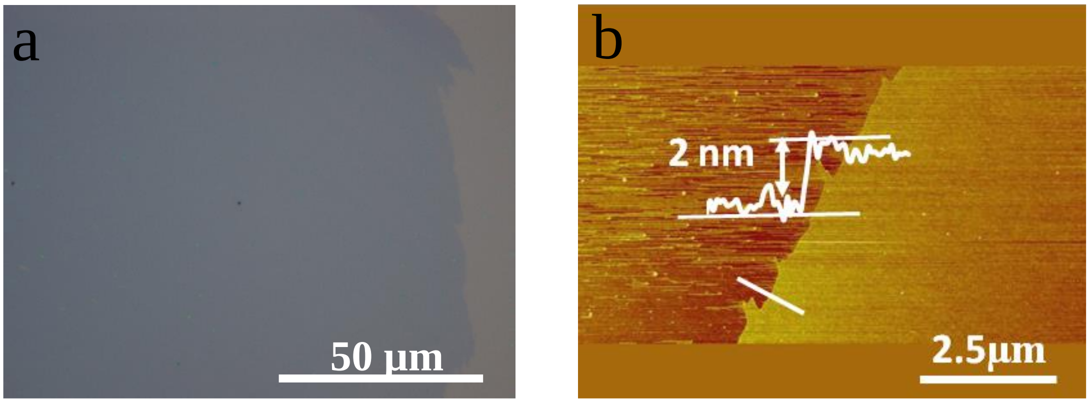

Supplementary Figure 4 | a, Optical and b, AFM images of MMC on bare SiO_2/Si substrate. We can see the MMC on bare SiO_2/Si substrate is very uniform and smooth. The height of the MMC is about 2 nm.

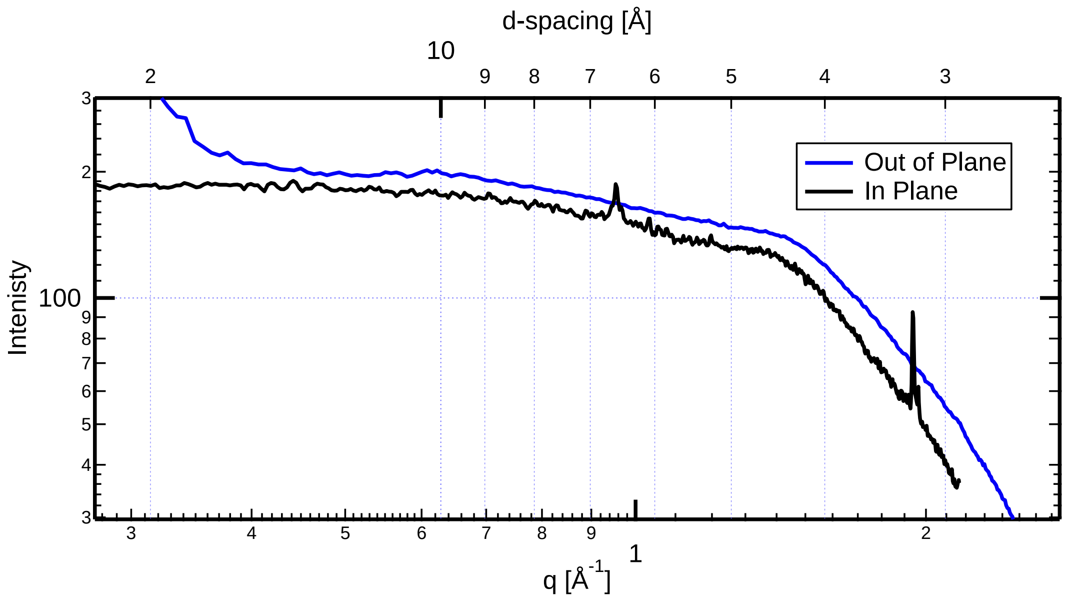

Supplementary Figure 5 | 1D plots corresponding to GIWAXS 2D scattering patterns of monolayer. The sharp in-plane lamella is 0.66 nm for the monolayer, evidence of strong in-plane ordering with long-range order, but the out of plane lamella is faint.

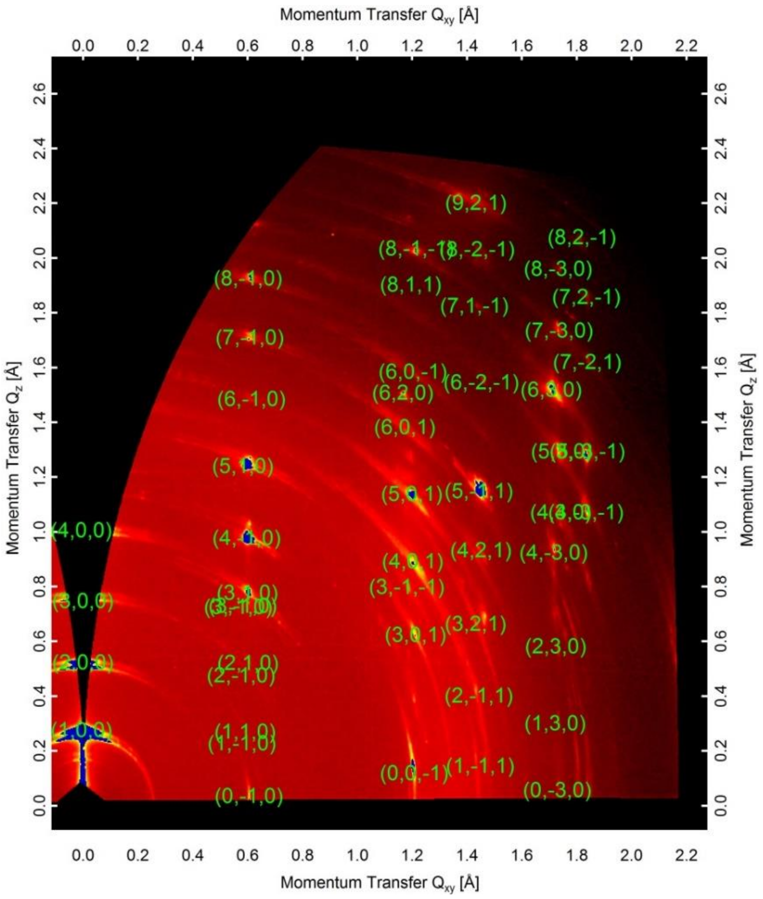

Supplementary Figure 6 | GIWAXS scattering pattern of the thick films of CMUT. The 2D GIWAXS scattering pattern of a thick film of CMUT. The GIWAXS pattern of a thick film is different from that of the monolayer, the optimized monoclinic lattice parameters are: a=2.54, b=1.12 nm, c=0.51 nm, α=75.6^o, β=85.3^o, γ=90.7^o, with the (h00) direction normal to the surface. No reflection corresponds to the 0.66 nm lamella spacing of the monolayer was seen, indicating that the first layer stacking is considerably different with that of the bulk crystal. Many areas of sample show only the h00 peaks, in accordance with the XRD theta-2theta scan results of thick films (Supplementary Figure 7), which indicates a layer-by-layer growth model. Here, a thick film of CMUT was prepared by drop casting method.

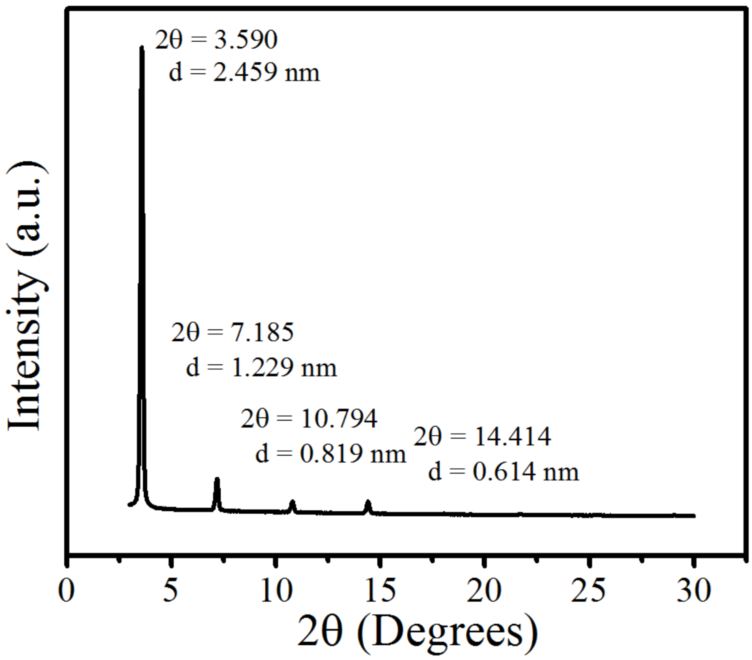

Supplementary Figure 7 | XRD theta-2theta scan results of CMUT thick films. We can see a series of multiple diffraction peaks. The *d* spacing is 2.459 nm, which approximates the GIWAXS pattern of thick films.

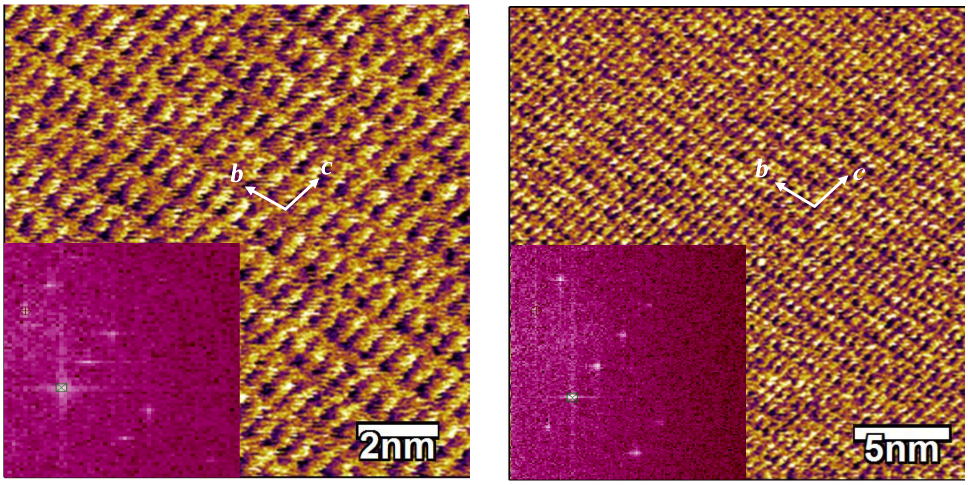

Supplementary Figure 8 | High-resolution AFM images and corresponding fast fourier transformation (FFT) of the thick films. Thick films are highly crystalline with typical brick-wall like packing in the (100) plane through FFT. Both *b* and *c* axes were marked. The lattice parameters of left and right panel are *b*=1.03 nm, *c*=0.53 nm, θ=108° and *b*=1.05 nm, *c*=0.51nm, θ=106°, which are different from that of monolayer.

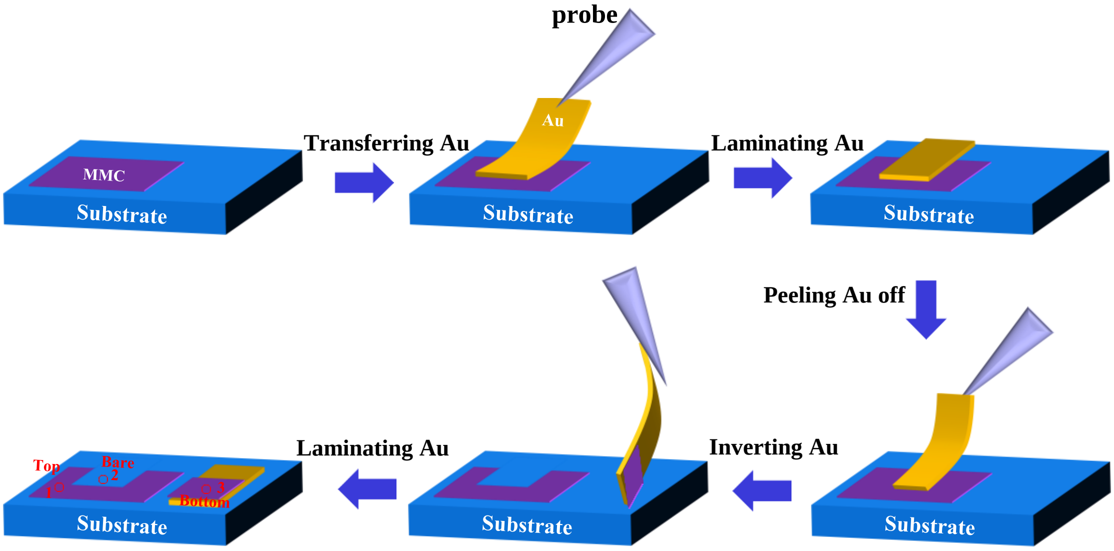

Supplementary Figure 9 | Schematic diagrams of preparation different sites on the same monolayer. In order to verify the monolayer growth and quasi-free-standing of the MMCs, we carried out HR-AFM study on the bottom of the MMCs. Firstly, an Au stripe was placed on the MMC with the probe and then lifted off. Three representative zones were shown in the left figure (bottom) in Supplementary Figure 9. Then three sites at the three zones were chosen for HR-AFM measurements.

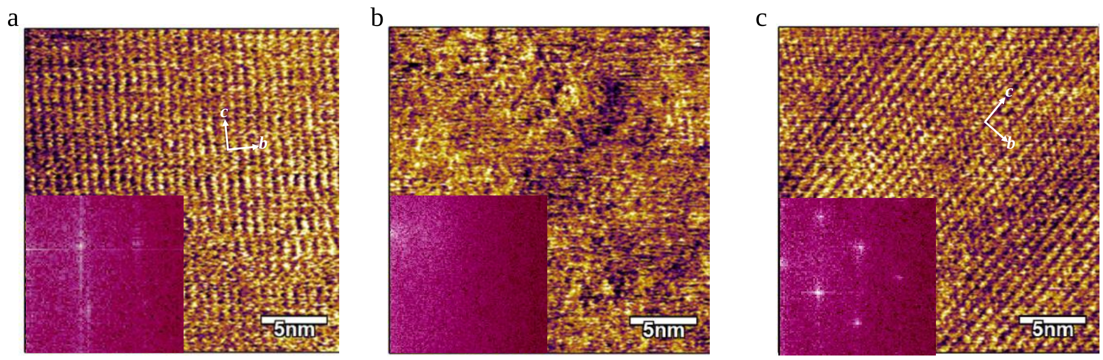

Supplementary Figure 10 | HR-AFM images of the top and bottom of MMCs. The preparation processes see Supplementary Figure 9. a, b, c, The HR-AFM measurements were carried at site 1, 2 and 3. The same lattice structures were found for MMCs on Au stripe (at site 3, b=0.84 nm, c=0.65 nm, θ=92°) as that on the original substrate (at site 1, b=0.83 nm, c=0.65 nm, θ=90°), and no lattice structures were found at on the substrate where the MMC was lifted off (at site 2). Both top side and bottom side of the MMCs exhibit indeed a very similar packing mode, demonstrating the neat MMCs on the substrates.

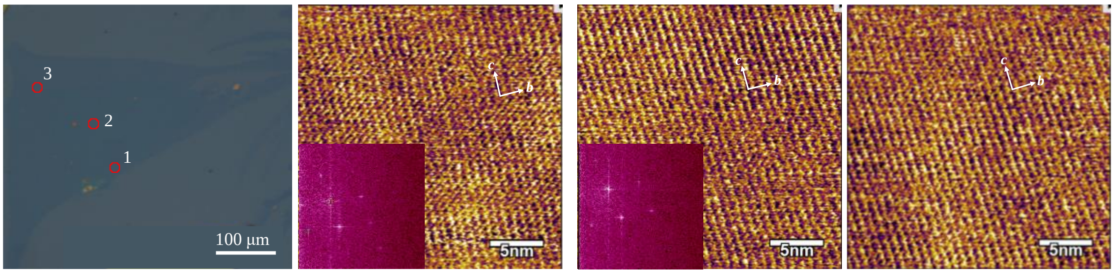

Supplementary Figure 11 | HR-AFM images of larger area of the monolayer. The HR-AFM measurements were carried at 1 (b=0.82 nm, c=0.63 nm, θ=88°), 2 (b=0.82 nm, c=0.63 nm, θ=91°) and 3 (b=0.82 nm, c=0.64 nm, θ=90°) sites. The same lattice structures were achieved at different sites, demonstrating that the large area MMC could be a single crystal. Here, the small angle of rotation of the lattice would be attributed to the inevitable rotation of the sample due to long-distance moving during the measurement processes.

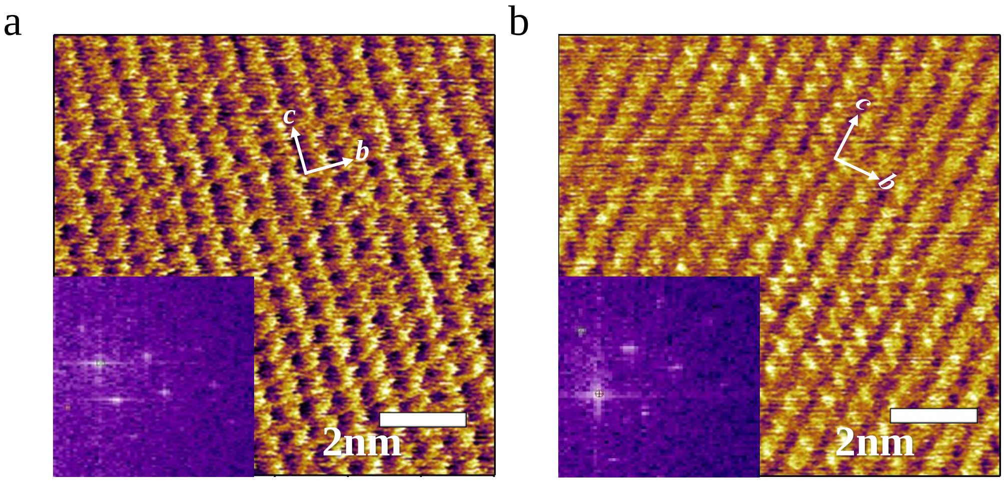

Supplementary Figure 12 | HR-AFM images of the MMC on SiO_2 and BCB substrates. High-resolution AFM images and corresponding FFT of the MMC **a,** on SiO_2 and **b,** BCB substrates. We found that both of the monolayer are highly crystalline with typical brick-wall like packing in the (100) plane through FFT. We can conclude that the lattices may be rectangle. Lattice parameters are b=0.83 nm, c=0.64 nm and θ= 90°, and b=0.84 nm, c=0.67 nm and θ=88° for a and b in monolayer respectively.

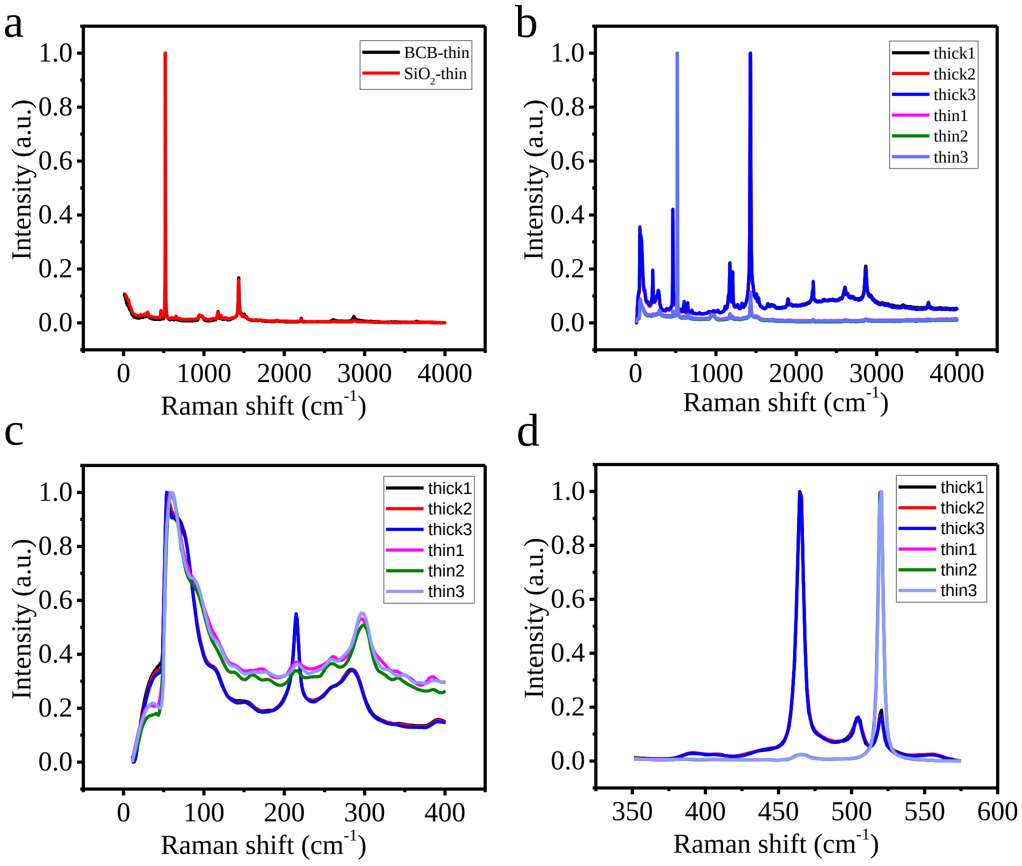

Supplementary Figure 13 | Raman spectrum of the MMCs and thick films. a, Normalized Raman spectrum of the MMCs on BCB and SiO_2 with 633 nm lasers (laser power 3.2%). The Raman peaks on BCB are identical with the MMCs on SiO_2. Combining with the HR-AFM measurements we demonstrate MMCs on BCB are the same with the MMCs on SiO_2. **b,** Normalized Raman spectrum of the MMCs and thick films on SiO_2 with 633 nm lasers (laser power 3.2%). **c** and **d,** typical peaks zoomed in **b**. The different peaks and Raman shifts confirm that the MMCs and thick films are in different phases.

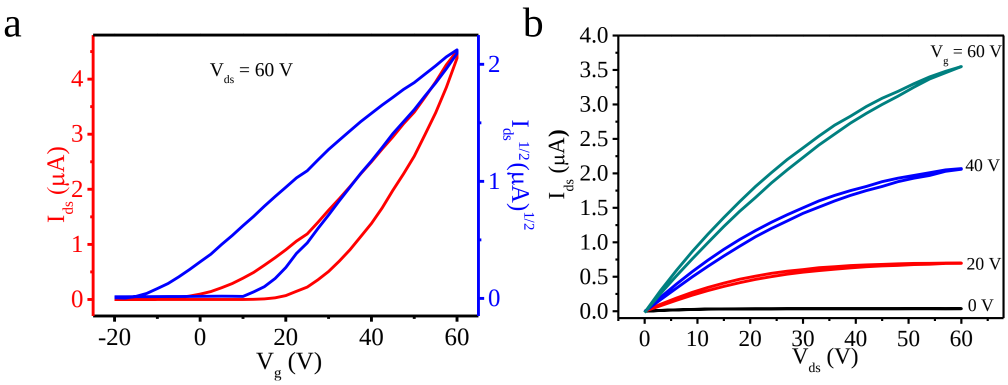

Supplementary Figure 14 | MMC FETs application. a, Transfer and **b,** output characteristic curves on bare SiO_2 substrate. The average electron saturation mobility is 0.12 $cm^2V^{-1}s^{-1}$ for the devices tested in ambient conditions.

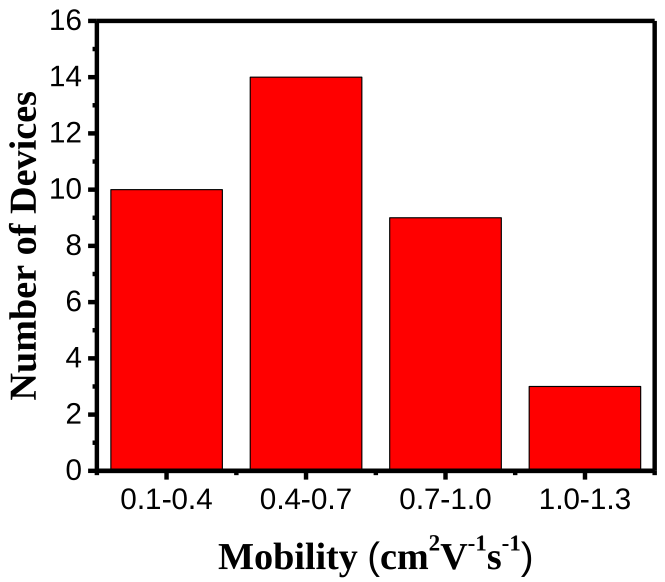

Supplementary Figure 15 | Statistics of the mobility values of MMC FET on BCB/SiO_2 substrates. The average saturation mobility value of the MMC FETs on BCB/SiO_2 substrates is about 0.5 $cm^2V^{-1}s^{-1}$ with maximum value up to 1.24 $cm^2V^{-1}s^{-1}$.

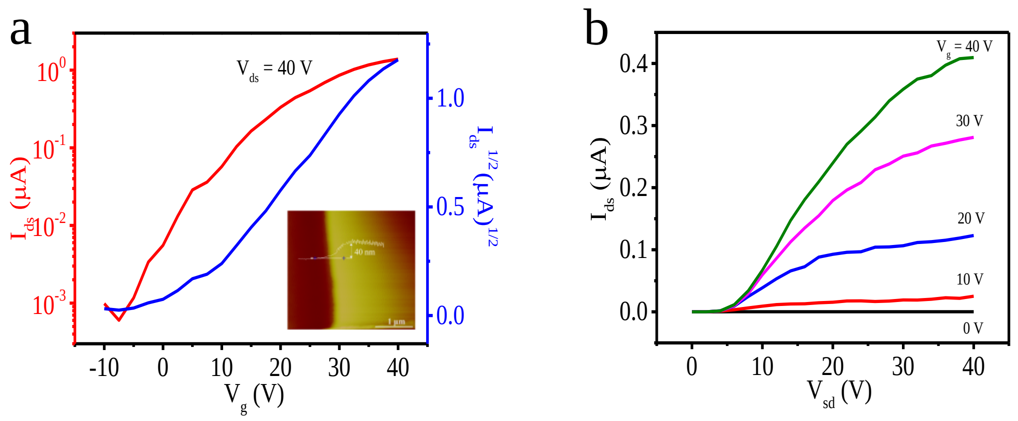

Supplementary Figure 16 | FETs application based on the thick crystalline films. **a,** Typical transfer and **b,** output curves of the thick films. The thicknesses of three samples are all about 40 nm. Inset of Supplementary Figure 16a is the AFM image of thick film in the corresponding device. The average electron saturation mobility is 0.39 $cm^2V^{-1}s^{-1}$ for devices tested in ambient conditions.

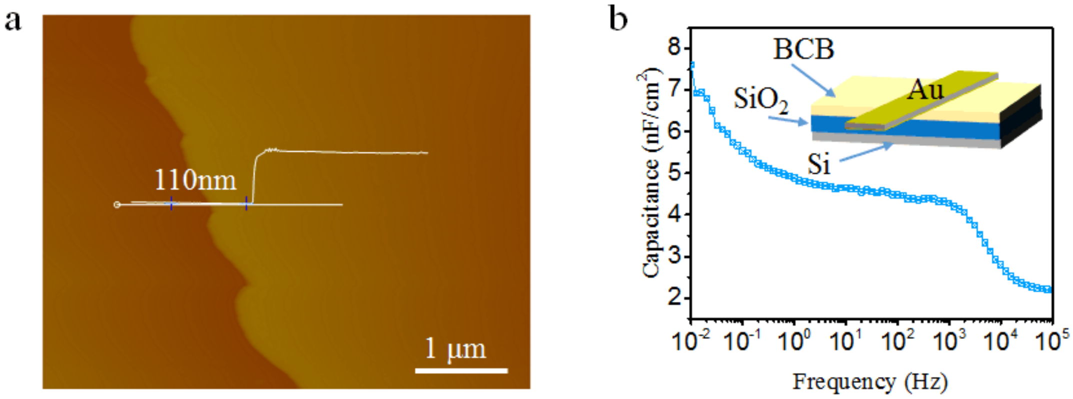

Supplementary Figure 17 | The evaluation of the capacitance of the BCB/SiO_2 dielectrics. The capacitance characteristic was measured with Princeton Applied Research. **a,** AFM image of spin-coated BCB with the thickness of 110 nm. **b,** Schematic structure of capacitor device with Au/110 nm BCB/300 nm SiO_2/Si configuration and capacitance characteristic of BCB/SiO_2 dielectric as a function of test frequency ranging from 10^4 to 0.01Hz, employing a capacitor with area of 0.16 cm^2.

In general, OFET characterization can be considered as a quasi-static process. For instance, a typical transfer characteristic sweeping cycle is commonly performed on a second scale (about 0.1 Hz). Hence, the corresponding capacitance characteristic for BCB/SiO_2 dielectric should be 5.53 nF/cm^2 (at 0.1 Hz) for the extraction of OFET mobility.

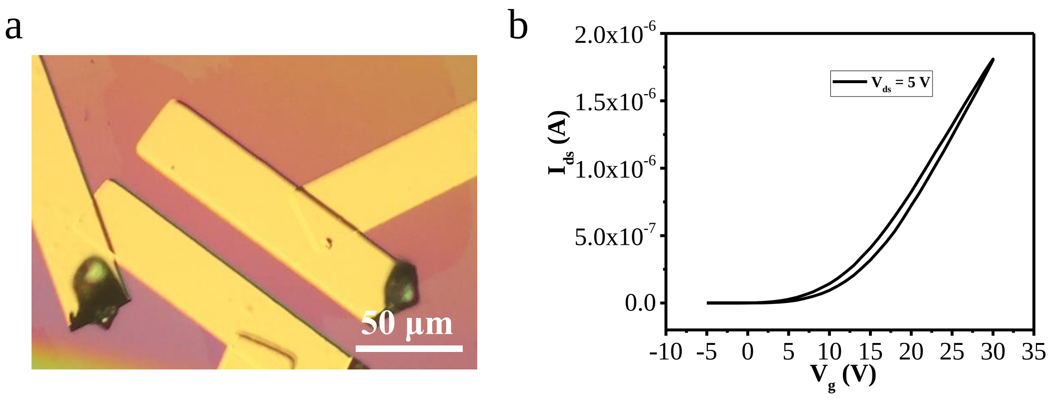

Supplementary Figure 18 | Typical optical microscopy image of MMC FET in Fig. 3a, b and corresponding linear transfer curve. a, Typical optical microscopy image of MMC FET. **b,** Transfer curves of this device in linear regime at V_{ds} = 5 V. (linear mobility is about 0.52 $cm^2V^{-1}s^{-1}$, which is close to the saturation mobility).

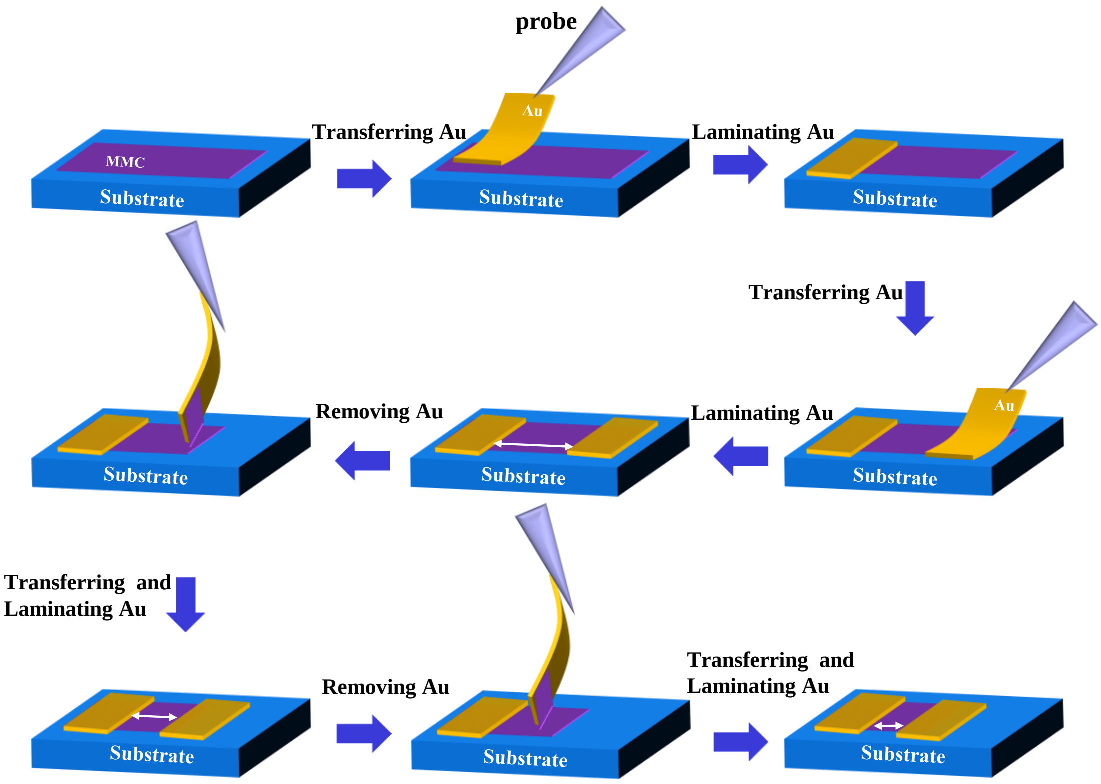

Supplementary Figure 19 | Schematic diagrams of preparing crystal FETs with different channel lengths. Firstly the bottom-gate and top-contact MMC/thick crystal FET was realized. After electrical property was measured, one Au electrode was held still, the other was peeled off and then another new Au stripe was laminated by cantilever probes for scaled down channel length of FETs. Repeating these procedures will produce crystal FETs with different channel length.

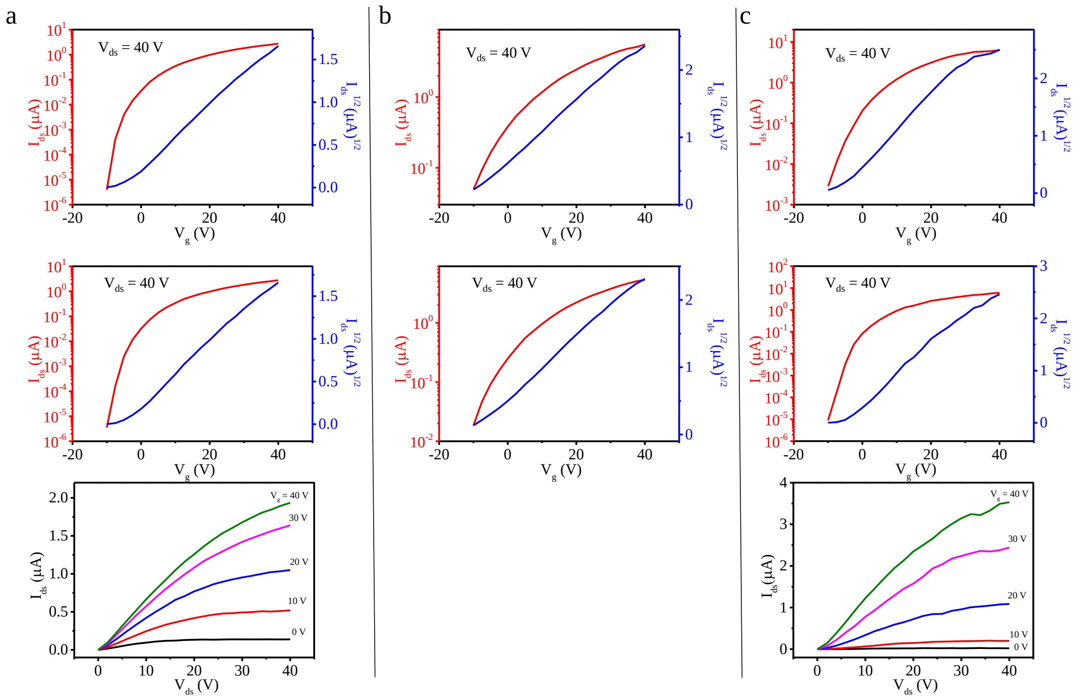

Supplementary Figure 20 | The electrical property characteristics of FETs at different channel lengths on BCB substrate. a, b, c, The transfer curves and output curves of FETs with channel length of about 31, 16 and 8 μm respectively. FETs with different channel lengths were realized by immobilizing one Au electrode and moving the other one (see Supplementary Figure 19). The device with channel length of 31 μm was firstly fabricated. Transfer curves were all measured twice to more accurately calculate the mobility. The mobility as a function of different channel length are mean values of two measurements. In devices with shorter channel, the current will slightly degrade after measurements of output curves due to higher current density, though the electrical property would be recovered several hours later. To avoid the effect and to probe the intrinsic channel length dependence of the mobility, we didn't measure the output curves of the device with medial channel length of 16 μm.

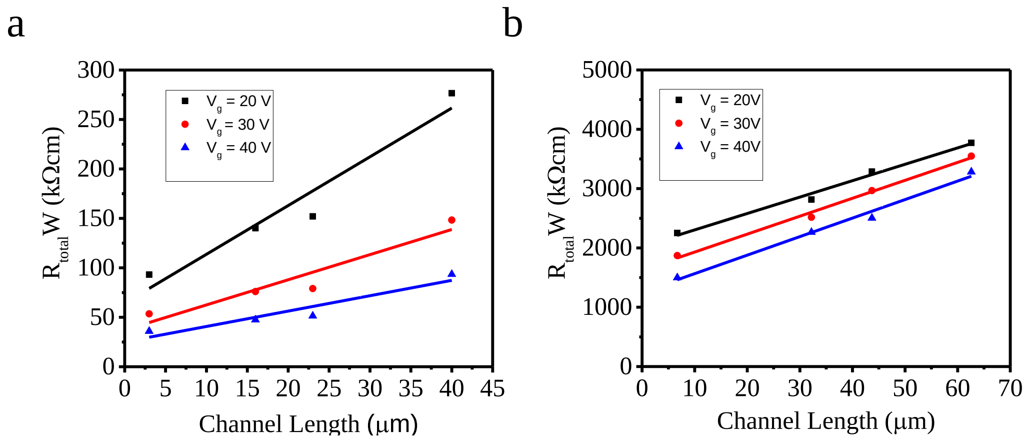

Supplementary Figure 21 | Contact resistance both in monolayer FETs and thick crystal FETs on BCB substrate. a, MMC FETs and **b,** FETs based on thick films with thickness of 40 nm at different gate voltages. By employing a conventional scaling analysis of the electrical characteristics of the FET devices at different channel length (Fabrication processes see Supplementary Figure 19), we extract the value of the contact resistance (R_C). The values of R_C in monolayer FETs are 64.4 kΩcm, 37.1 kΩcm, 25.2 kΩcm at Vg=20V, 30V, 40V respectively. While the values of R_C in thick crystal FETs are 2027.0 kΩcm, 1627.8 kΩcm, 1253.9 kΩcm at V_g=20V, 30V, 40V respectively. It demonstrated that the values of R_C in monolayer FETs are over 30 times smaller than that in the FET based on the thick crystals.

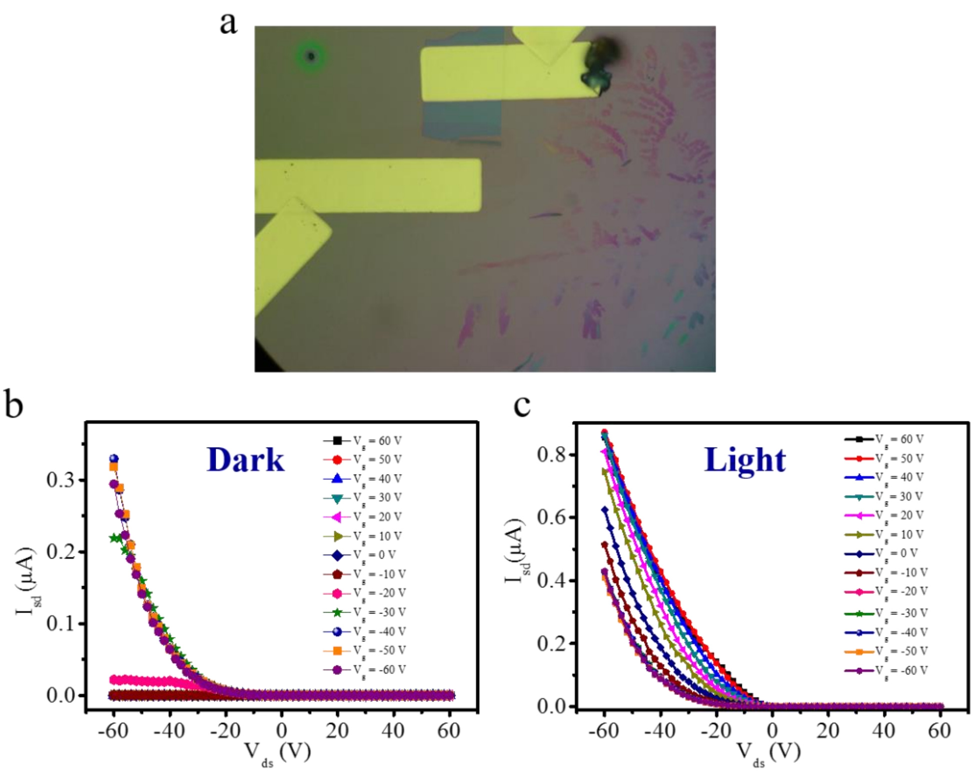

Supplementary Figure 22 | Optoelectronic property characterization of p-n diode.

a, Optical microscopy image of p-n diode. **b, c,** Output characteristic curves of the p-n diode (**b**) in the dark condition and (**c**) under light illumination at V_g = 60 ~ -60 V.

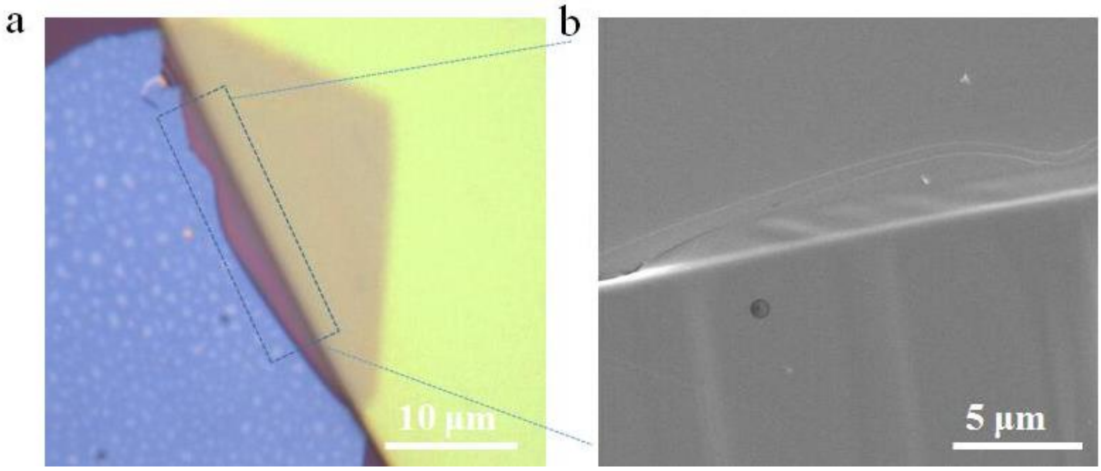

Supplementary Figure 23 | Realization of lateral p-n junction based on the thick film. a, Optical microscopy and **b,** SEM images of p-n junction based on the thick n-type film with thickness of 35 nm. The p-n junction was realized by laminating p-type single crystal DPA onto n-type MMC. DPA crystals are achieved by physical vapor transport (PVT) (165 ^{o}C, 5h, 20 Pa, under argon atmosphere (0.04 L/min)). The p-n junction was fabricated with cantilever probes to mechanically transfer crystalline DPA. Generally MMC was chosen but not thick crystalline films. When the thick crystalline films were used the p-type DPA cracked at the junction edge.

a b

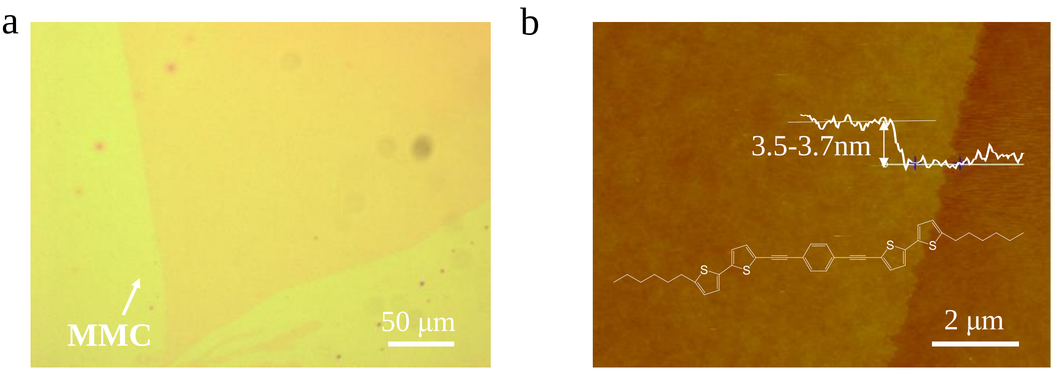

Supplementary Figure 24 | Another material of MMC prepared on BCB/SiO_2 substrates. **a,** Optical microscopy and **b,** AFM image of 1,4-bis((5’-hexyl-2,2’-bithiophen-5-yl) -ethynyl)benzene (HTEB).

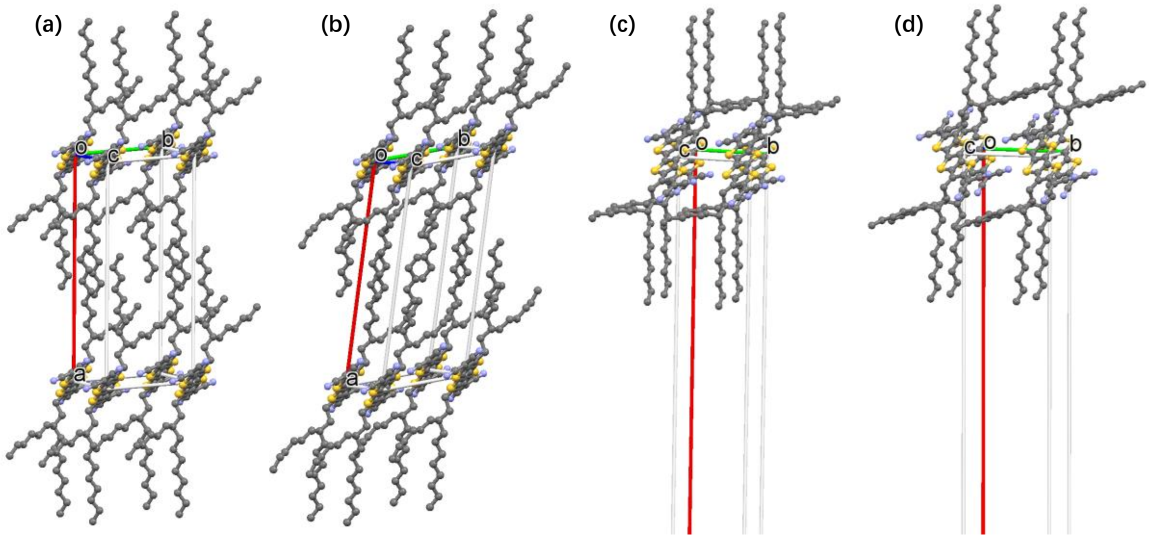

Supplementary Figure 25 | Bulk and monolayer crystal structures optimized by DFT-D3 with constrained and relaxed cell parameters. a, bulk (a=25.4Å, b=11.2Å, c=5.1Å, α=75.6º, β=85.3º, γ=90.7º), **b,** bulk-fullopt (a=24.8Å, b=10.6Å, c=5.1Å, α=73.5°, β=98.7°, γ=97.2°), **c,** monolayer (b=8.4Å, c=6.5Å, θ=90º), **d,** monolayer-fullopt (b=9.9Å, c=5.0Å, θ=101.6°).

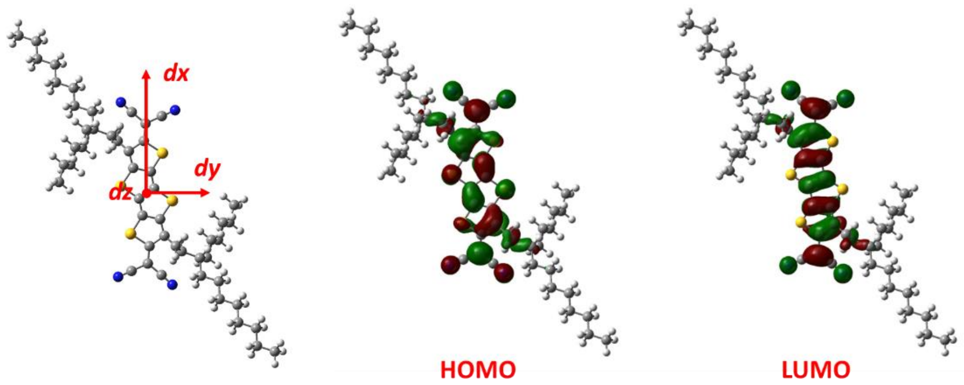

Supplementary Figure 26 | Illustration of the molecular long- and short-axes and the DFT-HSE06/def2-SVP calculated wave functions of the frontier orbitals of CMUT.

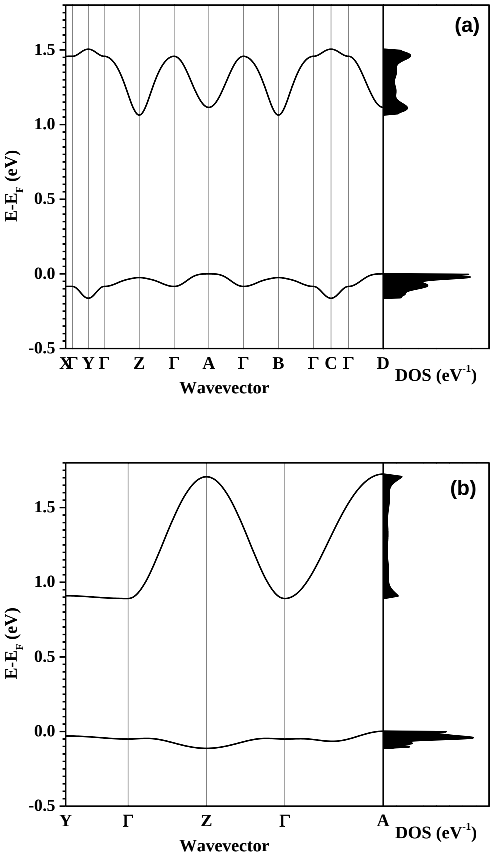

Supplementary Figure 27 | Band structures and density of states (DOS) for the bulk (a) and monolayer (b) crystals with the experimental cell parameters. The high-symmetry points in the first Brillouin zone are labelled as follows: Γ = (0,0,0), X = (0.5,0,0), Y = (0,0.5,0), Z = (0,0,0.5), A = (0,0.5,0.5), B = (0.5,0,0.5), C = (0.5,0.5,0), and D = (0.5,0.5,0.5), all in crystallographic coordinates. The Fermi energy is taken as the origin of the energy axis.

Supplementary Tables

	λ^-	λ^0	λ
HSE	163	146	309
B3LYP	166	150	316

Supplementary Table 1 | Reorganization energies (λ, in meV) for electron transfer of CMUT. λ^- represents the relaxation energy in the anionic state in going from the neutral to the anionic geometry, and λ^0 the relaxation energy in the ground state in going from the anionic to the neutral geometry.

	d	***dx***	***dy***	***dz***	***t* (HSE)**	***t* (B3LYP)**
**bulk**	5.10	3.11	2.27	3.34	96	102
**bulk-fullopt**	5.05	3.02	2.37	3.28	89	95
**monolayer**	6.70	5.75	1.12	3.25	204	214
**monolayer-fullopt**	5.04	3.28	1.99	3.27	147	154

Supplementary Table 2 | Electron transfer integrals (*t*, meV), center-of-mass distances, displacements along the molecular long- and short-axes, and perpendicular distances (*d*, *dx*, *dy*, and *dz*, in Å) for the *π*-*π* stacking molecular pairs taken from the simulated crystals.

	extreme	*m*	parallel to
**bulk**	B	0.84	c-0.17b
		4.09	b+0.30c
		∞	a-0.45c+0.08b
**bulk-fullopt**	D	1.19	c-0.06b
		7.20	b-0.37c
		∞	a+0.62c+0.21b
**monolayer**	Γ	0.35	c-0.03b
		16.71	b+0.01c
**monolayer-fullopt**	(0, 0.493, 0.5)	0.70	c+0.04b
		24.30	b+0.23c

Supplementary Table 3 | Effective masses for electrons (*m*, in unit of the electron mass at rest, m_0) at the band extremes of the simulated crystals.

Supplementary Notes:

Supplementary Note 1 | Computational details for transfer integrals and reorganization energies

The transfer integrals for the molecular pairs taken from DFT-D3 optimized crystal structures were evaluated by using a fragment orbital approach in combination with a basis set orthogonalization procedure.[2,3] To further understand the charge-transport properties, the reorganization energies of isolated molecules were calculated from the relevant points on the potential energy surface. The molecular geometries of the neutral and radical-anion states were optimized by DFT at the HSE06/def2-SVP level. For comparison, transfer integrals and reorganization energies were also calculated with the B3LYP functional. These DFT calculations were carried out with the Gaussian 09 program.[4]

Supplementary References:

1 Jiang, L. *et al.* Millimeter-sized molecular monolayer two-dimensional crystals. *Adv. Mater.* **23**, 2059-2063 (2011).

2 Senthilkumar, K. *et al*. Charge transport in columnar stacked triphenylenes: Effects of conformational fluctuations on charge transfer integrals and site energies. *J. Chem. Phys.* **119**, 9809 (2003).

3 Valeev, E. F. *et al*. Effect of electronic polarization on charge-transport parameters in molecular organic semiconductors. *J. Am. Chem. Soc.* **128**, 9882-6 (2006).

4 Frisch, M.J. *et al.* Gaussian 09 (Gaussian, Inc., Wallingford, CT, USA, 2009).
